# Accuracy of detection of high-grade cervical intraepithelial neoplasia using electrical impedance spectroscopy with colposcopy

**DOI:** 10.1111/1471-0528.12096

**Published:** 2013-01-04

**Authors:** JA Tidy, BH Brown, TJ Healey, S Daayana, M Martin, W Prendiville, HC Kitchener

**Affiliations:** 1 Department of Gynaecological Oncology, Royal Hallamshire HospitalSheffield, UK; 2 University of Sheffield, Royal Hallamshire HospitalSheffield, UK; 3 Department of Medical Physics, Royal Hallamshire HospitalSheffield, UK; 4 Department of Gynaecological Oncology, St Mary's HospitalManchester, UK; 5 ANP Women's Healthcare, Tallaght HospitalDublin, Ireland; 6 Department of Obstetrics & Gynaecology, Coombe Women's Hospital, Tallaght Hospital, Royal College of Surgeons in IrelandDublin, Ireland; 7 School of Cancer Studies and Enabling Sciences, University of Manchester, Manchester Academic Health Science CentreManchester, UK

**Keywords:** Cervical neoplasia, colposcopy, impedance spectroscopy, positive predictive value, sensitivity, specificity

## Abstract

**Objective:**

To determine if electrical impedance spectroscopy (EIS) improves the diagnostic accuracy of colposcopy when used as an adjunct.

**Design:**

Prospective, comparative, multi-centre clinical study.

**Setting:**

Three colposcopy clinics: two in England and one in Ireland.

**Population:**

Women referred with abnormal cytology.

**Methods:**

In phase 1, EIS was assessed against colposcopic impression and histopathology of the biopsies taken. In phase 2, a probability index and cut-off value for the detection of high-grade cervical intraepithelial neoplasia (HG–CIN, i.e. grade CIN2+) was derived to indicate sites for biopsy. EIS data collection and analyses were performed in real time and blinded to the clinician. The phase-2 data were analysed using different cut-off values to assess performance of EIS as an adjunct.

**Main outcome measure:**

Histologically confirmed HG–CIN (CIN2+).

**Results:**

A total of 474 women were recruited: 214 were eligible for analysis in phase 1, and 215 were eligible in phase 2. The average age was 33.2 years (median age 30.3 years, range 20–64 years) and 48.5% (208/429) had high-grade cytology. Using the cut-off from phase 1 the accuracy of colposcopic impression to detect HG–CIN when using EIS as an adjunct at the time of examination improved the positive predictive value (PPV) from 78.1% (95% CI 67.5–86.4) to 91.5%. Specificity was also increased from 83.5% (95% CI 75.2–89.9) to 95.4%, but sensitivity was significantly reduced from 73.6% (95% CI 63.0–82.5) to 62.1%, and the negative predictive value (NPV) was unchanged. The positive likelihood ratio for colposcopic impression alone was 4.46. This increased to 13.5 when EIS was used as an adjunct. The overall accuracy of colposcopy when used with EIS as an adjunct was assessed by varying the cut-off applied to a combined test index. Using a cut-off set to give the same sensitivity as colposcopy in phase 2, EIS increased the PPV to detect HG–CIN from 53.5% (95% CI 45.0–61.8) to 67%, and specificity increased from 38.5% (95% CI 29.4–48.3) to 65.1%. NPV was not significantly increased. Alternatively, applying a cut-off to give the same specificity as colposcopy alone increased EIS sensitivity from 88.5% (95% CI 79.9–94.4) to 96.6%, and NPV from 80.8% (95% CI 67.5–90.4) to 93.3%. PPV was not significantly increased. The receiver operator characteristic (ROC) to detect HG–CIN had an area under the curve (AUC) of 0.887 (95% CI 0.840–0.934).

**Conclusions:**

EIS used as an adjunct to colposcopy improves colposcopic performance. The addition of EIS could lead to more appropriate patient management with lower intervention rates.

## Introduction

The ability to identify correctly those who have, and those who do not have, disease is pivotal to the success of any screening programme. Prevention of cervical cancer depends on colposcopic detection and treatment of high-grade cervical intraepithelial neoplasia (HG–CIN, i.e. CIN2+) in women referred with abnormal cytology. Clinical performance of colposcopy is subjective and variable, dependent on factors including disease prevalence and training.^1^,^2^ Low colposcopic performance can result in failure to detect disease (inadequate sensitivity), or unnecessary treatment in the absence of disease (inadequate specificity).

Electrical impedance spectroscopy (EIS) can be used to identify tissue types. Determinants of impedance include cell layering, intra- and extracellular spaces, and the capacitance of the cell membranes. We have previously evaluated the ability of EIS to differentiate cervical tissues by developing a three-dimensional cellular model of the cervical epithelium. The models used a numerical analysis method often applied to the solution of physics field problems. This finite element modelling (FEM) involves modelling the tissue as elements (voxels) at the subcellular level, and including the features such as nuclear size and cell arrangements found in both normal and abnormal cervical epithelia.^3^–^6^

Colposcopy is reported to have the sensitivity and specificity to detect HG–CIN (CIN2+) ranging between 30–99 and 39–92%, respectively, with some studies showing a high sensitivity but low specificity and others a high specificity and low sensitivity.^7^ Recent and better quality studies have reported sensitivity of 56–60% and a positive predictive value of 60%.^8^–^10^ The wider range of values results in part because the literature contains the use of two methods to calculate sensitivity and specificity. In some reports the test standard used is a colposcopic impression of CIN2+, but in others the test standard used is an impression of disease being present and a biopsy being taken for the detection of any CIN2+ disease. The latter method leads to higher values of sensitivity but much lower values of specificity. If the disease threshold is defined as the worst biopsy showing HG–CIN or worse, then by definition the sensitivity will be 100%. In order to come closer to detecting all women with HG–CIN it would be necessary to take a very large number of biopsies. Cantor et al. 11 partially addressed this problem by taking some biopsies from one or two colposcopically normal sites per woman. The current study partially addresses the problem by using the biopsies suggested by both the colposcopists and EIS to define the group of women with high-grade disease. Cantor et al. used both definitions of the test standard in a large study and obtained a sensitivity of 71.4% and a specificity of 81.3% when the colposcopic appearances suggested high-grade squamous intraepithelial lesion (HSIL) or worse, and the histology of at least one of the biopsies indicated HSIL, but a sensitivity of 98.3% and a specificity of 45.1% when the colposcopic appearances suggested low-grade squamous intraepithelial lesion (LSIL) or worse, and the histology of at least one of the biopsies showed HSIL or worse.^11^

The primary objective of the study was to assess the ability of EIS when used as an adjunct to improve colposcopic performance, as measured by positive predictive value (PPV). The study was conducted in two phases. Phase 1 was designed to evaluate and validate the performance of the current EIS device against prior published data. We assessed performance before and after the application of 5% acetic acid to derive a cut-off value for the EIS measurement. The cut-off value per point was the median EIS measurement taken at sites of biopsy proven to be HG–CIN. In phase 2 we evaluated the performance of colposcopy with and without EIS, where the disease reference standard was defined as histologically confirmed HG–CIN in any biopsy suggested either by the colposcopist or by EIS, where the EIS measurement exceeded the median for HG–CIN derived from phase 1. In the UK the only published standard for colposcopic performance is the requirement for colposcopic impression of HG–CIN to exceed a PPV of 65%.^12^ This was taken as the gold standard for our trial, as there are no published data for sensitivity or specificity of colposcopy for the UK and Ireland.

The performance of colposcopy was determined first using a test standard of a colposcopic impression of CIN2+ and a biopsy taken in order to confirm this, and second using a test standard that disease may or may not be present at the time of colposcopic examination, but a biopsy was taken. Both these standards reflect normal colposcopic practice and guide subsequent clinical management. We also assessed the impact on colposcopic performance by varying the cut-offs for EIS when it is used as an adjunct to colposcopy.

## Methods

Phase 1 was performed at St Mary's Hospital, Manchester, UK, and the Royal Hallamshire Hospital, Sheffield, UK, and phase 2 included the Adelaide and Meath Hospital (AMNCH), Tallaght, Dublin, Ireland. Ethical approval for all sites and regulatory approval from the Irish Medicines Board (CI0021) and Medicines and Healthcare products Regulatory Agency (MHRA), UK, (CI/2008/0020) were obtained. All women referred with abnormal cervical cytology were eligible. Indication for referral for colposcopy followed the English and Irish cervical screening programmes. We expected that 37% of the study cohort would have high-grade cytology to reflect the published data.^13^ Exclusion criteria were pregnancy and active menstruation. Referrals were non-consecutive, and one British Society for Colposcopy and Cervical Pathology (BSCCP)-certified colposcopist at each centre performed all of the examinations. Cytology was reported in accordance with the screening programmes for England and Ireland. Histopathology was reported by specialist gynaecological pathologists at all centres, in accordance with local practice. Colposcopic examination was performed according to local practice and all colposcopic examinations were video recorded. Follow-up of women with no evidence of CIN, based on colpsocopic examination and biopsy results, followed local practice and national guidance.

Using information from our previous studies,^3^,^5^ and from Mitchell et al.,1998 we estimated that EIS data from 100 women would be sufficient to demonstrate an increase in PPV from 63.3 to 73.4%, for colposcopic impression used to detect HG–CIN. This calculation assumed a disease rate of 50% in the study population. Because PPV is dependent on the study population, which varies between the three referral centres, we chose to recruit 200 women to both phases of the study, in order to be confident of having sufficient statistical power to show an absolute increase of 10% in PPV.

### EIS measurements

**Figure 1:**
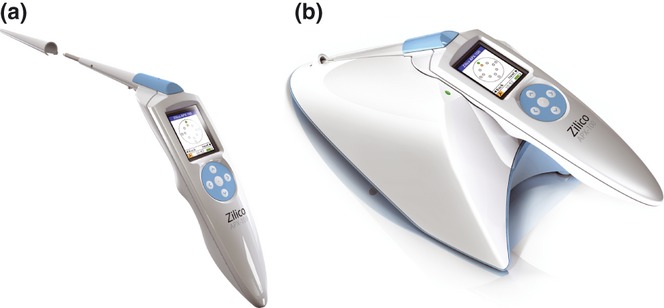
The EIS measurement device. (A) Hand-held unit with single-use disposable sheath. (B) Hand-held unit on base station. The device is operated via a small mobile phone-type display screen and toggle buttons mounted in the handle of the device.

The EIS device (Figure 1) consists of a hand-held unit, a base station for downloading data to a laptop, a disposable single-use sheath covering the snout of the hand-held unit and associated software. The 5.5-mm diameter tip of the snout is placed in contact with the cervix, as described in our earlier publications.^3^–^5^ At the tip of the sheath are four gold electrodes, the diameter and spacing of which determine the flow of current into the cervical tissue.

A four-electrode transfer impedance measurement is made. The current (<12 μA p–p) is injected between an adjacent pair of electrodes, and voltage is measured between the remaining pair. The frequency of the applied current ranges from 76.3 to 625 kHz in 14 steps. The minimum time required to record the full frequency spectrum is 20 milliseconds. Measurements are made having passed five quality control assessments and a cut-off for tissue homogeneity across the four electrodes. Data is captured in real time on the device and downloaded to the laptop for data analysis.

In phase 1, 12 colposcopically guided EIS measurements were taken from the cervix: ten from the squamocolumnar junction and two at the 12 and 6 o'clock positions on native squamous epithelium, away from the transformation zone. After the application of acetic acid measurements were repeated, but with no intention to take both sets of readings from exactly the same sites. Colposcopy was performed and biopsies were taken as clinically indicated. Video review was undertaken to assign a colposcopic impression for every reading site and to assess the co-location of EIS sites with biopsies. An overall colposcopic opinion was given for each site, taking into account colposcopic impression and histological data. Normal colposcopic examinations were not validated by taking random biopsies because of the high negative predictive value of colposcopy in this situation, and the taking of such biopsies is not part of current UK or Irish colposcopy practice.^14^–^16^ All videos in phase 1 were reviewed by the chief investigator to check for consistency between centres and with the methodology used in our previous publications.^3^–^5^,^17^ We evaluated the performance of EIS on a per-point basis, using final colposcopic opinion to separate HG–CIN from all other tissue types, for comparison with our earlier publications.^3^–^5^ The per-point data were used to determine a cut-off for the detection of HG–CIN, based upon biopsies taken from the points where EIS measurements were taken. This cut-off was then used in phase 2 of the study.

In phase 2, 12 EIS measurements were made after the application of acetic acid but prior to the formal colposcopic examination. All EIS measurements were downloaded to the laptop running EIS software and the colposcopist then indicated any clinically chosen sites for biopsy by highlighting, on an input screen, the EIS measurement site corresponding to the proposed biopsy site. The software analysed the EIS data and displayed any additional sites that were above the cut-off, where HG–CIN was probable. The EIS results for any biopsies chosen by the clinician were not displayed so that these were independent of the use of EIS. The clinician was requested to biopsy additional sites but was not mandated to take any additional EIS-directed biopsies. A maximum of three biopsies per patient, clinical and EIS combined, was suggested, but could be exceeded on clinical indication. A contemporaneous colposcopic impression for all biopsies was recorded. Phase 2 videos were reviewed by the colposcopist at each study site, allowing the evaluation of concordance between EIS measurements and biopsy sites.

Prior experience with the EIS devices suggested a learning curve of up to 20 examinations to gain expertise with the device and study protocol. Completion of this training phase was assessed on the technical quality of the EIS readings and by a review of the colposcopic examinations by the colposcopist and the chief investigator.

### Methods of analysis

The EIS spectra were compared with templates corresponding to normal squamous and columnar epithelia, immature metaplasia and HG–CIN. Templates were generated from three-dimensional finite element models of the four tissue types, as described by Walker et al.^6^,^18^ Matches between EIS spectra and templates were made using a minimum least squares method. Biopsy results from phase 1 and measured EIS spectra were used to construct probability distributions for each tissue type, enabling the absolute probability that a measured EIS spectrum corresponded to HG–CIN, and also the relative probability that the measurement corresponded to the other tissue types, to be determined. Both probabilities were used to determine a probability index (PI), which was given a value between zero and one, for the detection of HG–CIN. The median value of PI for biopsy-proven HG–CIN measured in phase 1 was set as the cut-off to suggest additional points for biopsy in phase 2. At the conclusion of phase 2 of the study the PI was calculated for every measured spectrum and linked to the final colposcopic opinion. When evaluating the use of EIS as an adjunct to colposcopy, the colposcopic impression (CI) per point was combined with PI, colposcopic impression was scored as 1 if HG–CIN was thought to be present but zero if HG–CIN was thought to be absent, and the two results were summed. This resulted in a combined probability index (CI + PI) with a value between zero and two. Because PI is a continuous variable and because both it and CI were recorded for all points in each woman, it was possible to analyse the performance of the (CI + PI) index, not only for the cut-off used to suggest the additional points for biopsy, but also for other cut-offs.

For each woman the worst (CI + PI) result was returned as the test result. The worst biopsy result from all the biopsies taken was used to define the disease category for the woman. Using these data the effect of varying the cut-off to detect HG–CIN was evaluated using receiver operator characteristic (ROC) analysis to assess the performance of EIS on its own and as an adjunct to colposcopy. The area under the curve (AUC) was calculated to measure the ability to separate women with and without HG–CIN.

Sensitivities and specificities for clinical performance alone were calculated in the two ways discussed in the Introduction. These will be referred to as colposcopic impression (CI) and disease present (DP). In the first method (CI), the test result was positive if HG–CIN was thought to be present and a biopsy was taken for confirmation. In the second method (DP), the test result was positive if it was thought that some disease was present and hence a biopsy was required to confirm or exclude HG–CIN. In both cases data were analysed on a per woman basis reflecting the usual clinical decision-making process. For CI the test result was counted as positive if any of the 12 EIS measurement points was considered as HG–CIN on colposcopic examination. For DP the test result was counted as positive if at least one EIS measurement point was suggested for biopsy. In both cases a woman was counted positive for HG–CIN if any of the biopsies, suggested either by the clinician or by EIS, confirmed HG–CIN. The results for colposcopy with EIS as an adjunct used the highest CI + PI value.

When applying the CI + PI method referral cytology was taken into account: a higher PI cut-off for HG–CIN was used for low-grade cytology compared with high-grade referrals to minimize the possibility of false-positive results and unnecessary biopsies. The different CI + PI cut-offs for referral cytology were maintained in all the analyses.

The ROC curves were plotted using matlab™ (The MathWorks Inc., Natick, Massachusetts, USA). *P* values were calculated by comparing colposcopy with and without AXP100 using a two-tailed non-parametric *Z*-test analysed in prism 5.04 (GraphPad Software Inc., La Jolla, California, USA).

## Results

Four hundred and seventy-four women were recruited to the study between April 2009 and May 2011: 247 women in phase 1 and 227 women in phase 2. Thirty-three women in phase 1 were excluded: 31 as part of training and two because of incomplete clinical data. Twelve women were excluded in phase 2: nine had incomplete clinical data, one did not meet the inclusion criteria, one was unable to complete the colposcopic examination and one was excluded because of a protocol violation. In five cases the device exhibited technical problems that prevented the collection of EIS data. Additionally, 110/7706 (1.4%) recorded measurements were unacceptable when the spectra were visually reviewed. The demographics of the 429 eligible women are presented in Table 1.

**Table 1:** Demographic and referral smear data for women recruited into phases 1 and 2

	Phase 1	Phase 2
**Demographic**	*n* = 214 (100%)	*n* = 215 (100%)
Median age (years)	31.3	29.5
Age range (years)	20–60	20–64
Postmenopausal (%)	7 (3.3)	9 (4.2)
**Ethnicity (%)**		
White	195 (91)	194 (90)
Indian/Asian	4 (2)	7 (3)
African/Black	10 (5)	12 (6)
Oriental	4 (2)	1 (0)
Other	1 (0)	1 (0)
**Referral cytology (%)**		
Borderline	48 (22.4)	58 (27)
Borderline glandular	1 (0.5)	0 (0)
Mild dyskaryosis	52 (24.3)	63 (29.3)
Borderline, high-grade not excluded*	7 (3.3)	13 (6)
Moderate dyskaryosis*	32 (15)	23 (10.7)
Severe dyskaryosis*	71 (33.2)	49 (22.8)
Invasive*	1 (0.5)	1 (0.5)
Glandular neoplasia*	2 (0.9)	8 (3.7)
**Colposcopy clinic (%)**		
Sheffield	159 (74.3)	76 (35.3)
Manchester	55 (25.7)	68 (31.6)
Dublin	0 (0)	71 (33.1)

1 Classified as high-grade cytology referrals.

In phase 1, 52.8% (113/214) of women had a high-grade cytology referral; in phase 2, 43.7% (94/215) of women had a high-grade cytology referral. The mean number of directed biopsies over all women was 1.42 per woman in phase 1 (range 0–4) and 1.68 in phase 2 (range 0–4). Where a biopsy was taken the mean results were 1.71 (range 1–4, 178 out of 214 women) and 1.77 (range 1–4, 186 out of 196 women).

Two adverse events and one serious adverse event were reported. One patient felt unwell and two had problems with bleeding after biopsies.

### Phase 1

The ROC analysis of phase 1 EIS per-point data resulted in an AUC of 0.783 (95% CI 0.755–0.812). EIS performance was unaffected by the use of acetic acid. The median value of the PI for the detection of HG–CIN was 0.568. This cut-off was used to suggest the additional biopsies that were taken in phase 2. The median cut-off will include 50% of the points with an HG–CIN biopsy result. However, because up to 12 points were assessed per woman and the results were used as an adjunct with colposcopic impression, this will not limit the per-woman sensitivity to 50%. In addition, because EIS and CI data were recorded for all points it was possible to vary the threshold in the subsequent analysis of all the phase 2 data.

### Phase 2

**Figure 2:**
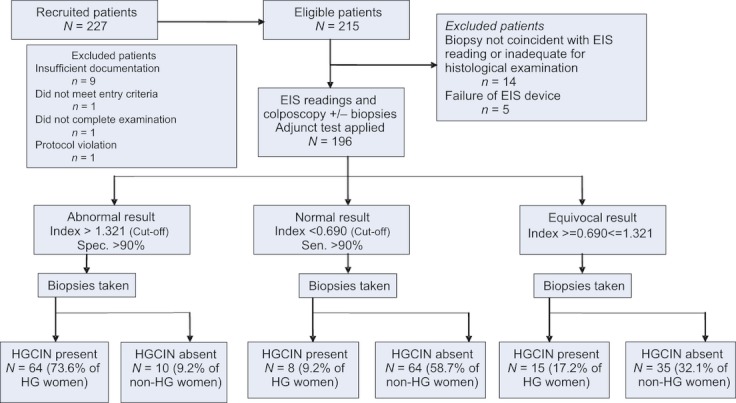
Flowchart of women in phase 2 of the study. The results are presented based upon a per woman analysis using the CI + PI method. There were 87 women with HG–CIN and 109 women with non-HG–CIN, as judged by pathology results from all of the biopsies taken.

Figure 2 illustrates the flow of women who were recruited in phase 2: 196 were evaluated using EIS as an adjunct to colposcopy. Sixty-seven (75.2%), of the 87 women referred with high-grade cytology, and 20 (18.6%) of the 109 women referred with low-grade cytology, had biopsy-proven HG–CIN.

The performance of colposcopy using colposcopic impression for HG–CIN (CI) is shown in Table 2: sensitivity 73.6%; specificity 83.5%; PPV 78.1%; NPV 79.8%. When the DP method was used the baseline performance for colposcopy was: sensitivity 88.5%; specificity 38.5%; PPV 53.5%; NPV 80.8% (Table 3).

**Table 2:** Relative performance using colposcopic impression alone to detect HG–CIN (CI method) and colposcopic impression combined with EIS to predict the presence of HG–CIN

*n* = 196	Colposcopic impression Cut-off = HG–CIN	Colposcopic impression + EIS (CI + PI) Cut-off = 1.321^*^	Statistical significance	Colposcopic impression + EIS (CI + PI) Cut-off = 1.083^**^	Statistical significance	Colposcopic impression + EIS (CI + PI) Cut-off = 1.568^***^	Statistical significance
Sensitivity	73.6% (95% CI 63.0–82.5)	73.6%	NS	78.2%	NS	62.1%	*P* = 0.0394
Specificity	83.5% (95% CI 75.2–89.9)	90.8%	*P* = 0.0226	83.5%	NS	95.4%	*P* = 0.0010
PPV	78.1% (95% CI 67.5–86.4)	86.5%	*P* = 0.0456	79.1%	NS	91.5%	*P* = 0.0012
NPV	79.8% (95% CI 71.3–86.8)	81.2%	NS	82.7%	NS	75.9%	NS
Positive likelihood ratio	4.46 (95% CI 3.17–7.73)	8.00	*P* = 0.0308	4.73	NS	13.50	*P* < 0.0001

The cut-off shown is that applied to the CI + PI probability index. It corresponds to an initial clinical opinion that HG–CIN was present or not and a probability index of 0.321^*^, 0.083^**^ or 0.568^***^ derived from the EIS measurements. The statistical significance tests the CI + PI method at the specified cut-offs in comparison with colposcopic impression alone, as expressed using the CI method.

**Table 3:** Relative performance of colposcopy alone, based on evidence of disease such that a biopsy was suggested, the DP method and colposcopy combined with EIS to detect the presence of HG–CIN

*n* = 196	Colposcopy cut-off = any biopsy taken	Colposcopic impression + EIS (CI + PI) Cut-off = 0.768^*^	Statistical significance	Colposcopic impression + EIS (CI + PI) Cut-off = 0.390^**^	Statistical significance	Colposcopic impression + EIS (CI + PI) Cut-off = 0.568^***^	Statistical significance
Sensitivity	88.5% (95% CI 79.9–94.4)	88.5%	NS	96.6%	*P* = 0.0060	92.0%	NS
Specificity	38.5% (95% CI 29.4–48.3)	65.1%	*P* < 0.0001	38.5%	NS	51.6%	*P* = 0.0076
PPV	53.5% (95% CI 45.0–61.8)	67.0%	*P* = 0.0006	55.6%	NS	60.3%	NS
NPV	80.8% (95% CI 67.5–90.4)	87.7%	NS	93.3%	*P* = 0.0094	89.0%	NS
Positive likelihood ratio	1.43 (95% CI 1.24–1.69)	2.53	*P* < 0.0001	1.57	NS	1.90	*P* = 0.0002

The cut-off shown is that applied to the CI + PI probability index. It corresponds to an initial clinical opinion that HG–CIN was present or not, and a probability index of 0.768^*^, 0.390^**^ or 0.568^***^ derived from the EIS measurements. The statistical significance tests the CI + PI method at the specified cut-offs in comparison with colposcopy alone, as expressed using the DP method.

The results were analysed using the cut-off of 0.568 from phase 1 that was used to suggest the additional biopsies in phase 2. The accuracy of colposcopic impression to detect HG–CIN when using EIS as an adjunct at the time of examination (Table 2) improved the PPV from 78.1% (95% CI 67.5–86.4) to 91.5%. Specificity was also increased from 83.5% (95% CI 75.2–89.9) to 95.4%, but sensitivity was significantly reduced from 73.6% (95% CI 63.0–82.5) to 62.1%, and the negative predictive value (NPV) was unchanged.

**Figure 3:**
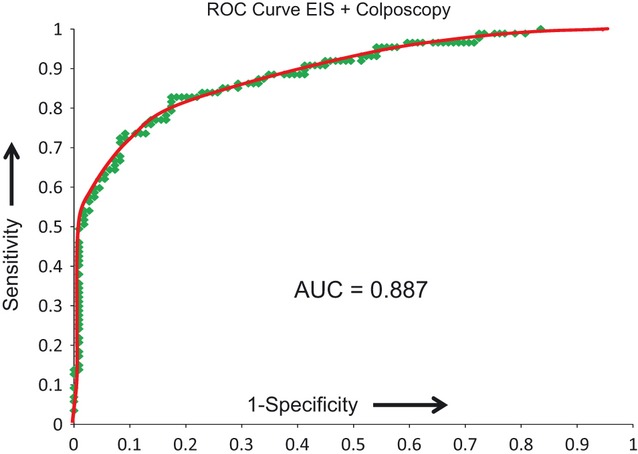
Receiver operator characteristic curve for colposcopy with EIS as an adjunct using the CI + PI method. This was produced using data from all the women in phase 2 and by varying the threshold applied to the CI + PI index.

Given the lack of data on colposcopic performance, we were unsure if colposcopy was over performing in our study compared with the published data. On pragmatic grounds we thought that colposcopists may not wish to trade an improved PPV and specificity for a loss in sensitivity; therefore, we conducted further analysis of the data. By varying the cut-off applied to the CI + PI index we could further assess the performance of EIS, as an adjunct to colposcopy. By selecting a high cut-off, high specificity but low sensitivity are achieved, and vice versa. Using this approach we produced an ROC curve for EIS as an adjunct to colposcopy to detect HG–CIN (Figure 3), with an area under the curve of 0.887 (95% CI 0.840–0.934). This curve uses the results for all the women in phase 2. For the purposes of discussion we also separated the data into three groups using the CI + PI method: one above a high cut-off (1.321), indicating general agreement between the colposcopist and EIS on the presence of HG–CIN; one below a low cut-off (0.69), where there was agreement on the absence of HG–CIN; and finally a third group where the data were equivocal. The cut-off between the three groups was chosen to correspond to a sensitivity of 90% and specificity of 90% for the abnormal and normal groups, respectively. The equivocal group contained 15 women with biopsy-proven HG–CIN (seven with high-grade and eight with low-grade cytology referrals) and 35 women with no evidence of HG–CIN (six with high-grade and 29 with low-grade cytology referrals). Using the data from EIS led to seven extra women with HG–CIN (8.0% of the women with proven HG–CIN) being detected in this group that had been missed by the colposcopist. There were eight cases of HG–CIN (9.2% of the women with proven HG–CIN) that were detected by colposcopy but missed by EIS. Of the 35 women with no proven evidence of HG–CIN, 25 (22.9% of all the women with no proven HG–CIN) were positive on EIS and ten (9.2% of all the women with no proven HG–CIN) were positive on colposcopy.

An ROC curve was calculated for the use of the EIS data alone, using information on the referral smear to set the cut-offs but not using colposcopic impression. The AUC for EIS alone was 0.827 (95% CI 0.770–0.885), the sensitivity was 86.2% (95% CI 77.1–92.7) and the specificity was 56.0% (95% CI 46.1–65.5) when using a cut-off of 0.568. PPV was 61.0% and NPV was 83.6%. The positive likelihood ratio was 1.96. This was significantly greater (*P* < 0.0001) than the figure of 1.43 for colposcopy alone when calculated using the DP method (Table 3).

The two methods of quantifying the performance of the colposcopists, CI and DP, are presented in Tables 2 and 3. In both cases these performance figures are compared with the performance of the CI + PI method. When using EIS as an adjunct to colposcopy and a CI + PI cut-off of 1.321, chosen to leave the sensitivity unchanged at 73.6%, both specificity and PPV are significantly increased compared with colposcopy alone. NPV is not significantly increased. Table 3 compares the results of the CI + PI method with the clinical performance as measured by the DP method. When using EIS as an adjunct to colposcopy and using a cut-off of 0.768, chosen to leave the sensitivity unchanged at 88.5%, both specificity and PPV are again significantly increased compared with colposcopy alone. NPV is also increased, but not at a significant level.

The cut-off applied to the CI + PI index can also be selected to leave specificity unchanged when using EIS as an adjunct to colposcopy. Using a cut-off of 1.083 the specificity, in comparison with clinical performance measured using the CI method, is unchanged at 83.5%. Sensitivity, PPV and NPV are all increased slightly, but not at a significant level (Table 2). When using a cut-off of 0.390 the specificity, in comparison with clinical performance measured using the DP method, is unchanged at 38.5%, but sensitivity is significantly increased from 88.5% (95% CI 79.7–94.4) to 96.6%, and NPV from 80.8 (95% CI 67.5–90.4) to 93.3% (Table 3). PPV is not significantly increased. The 2 × 2 tables corresponding to cut-offs of 0.568 and 0.768 for the DP method are given in Table 4. The figures for both colposcopy alone and when using EIS as an adjunct are also given.

**Table 4:** (A) A 2 × 2 table showing the true- and false-positive (TP and FP) and the false- and true-negative (FN and TN) numbers for a threshold of 0.568 when using the DP method of analysis. (B) A 2 × 2 table showing similar results, but using a threshold of 0.768. In both tables the figures in brackets are those for clinical colposcopy without EIS as an adjunct. Note that there are small differences between the sensitivity and specificity values derived from these numbers and the sensitivity values given in Table 3. This is because the ROC curve used to derive the values in Table 3 was a smoothed curve through the values

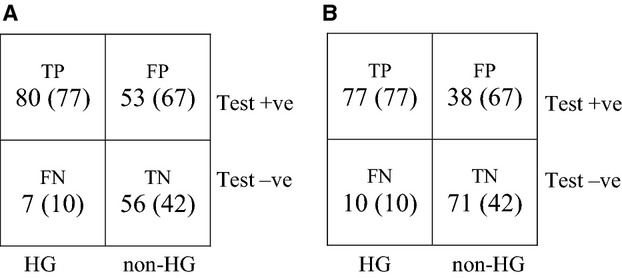

We calculated the positive likelihood ratio (LR+) for all the previously described cut-offs. LR+ for colposcopic impression was 4.46; in two of the three cut-offs the additional use of EIS significantly increased LR+ (Table 2). LR+ for disease present as detected by any colposcopically directed biopsy was 1.43, and again the addition of EIS significantly improved LR+ for two of the three cut-offs (Table 3).

## Discussion

This study shows that the use of EIS as an adjunct to colposcopy can improve the accuracy of disease detection, and the increase in PPV exceeded the objective of the study. By adjusting the cut-off applied to the EIS measurement specificity or sensitivity can be significantly improved in comparison with colposcopy alone. The LR+ is the likelihood of a given test result in a woman with disease, compared with the likelihood of getting the same result in a woman without the disease. The LR+ for colposcopic impression alone was 4.46, and this increased significantly to between 8 and 13.5 when using EIS as an adjunct (Table 2). Cantor et al. quote a figure of 3.81 for colposcopic impression, although their methodology was slightly different, as discussed in the Introduction.^11^ An LR+ > 8 indicates at least a 40% increase in the probability of disease being present.

Colposcopy plays a major role in the management of women with abnormal cervical cytology. Women with HG–CIN are offered treatment, and those with no abnormality can be reassured and returned to routine screening. Inaccurate colposcopy can result in diagnostic delay, over-treatment and unnecessary repeated colposcopic examinations. Colposcopic impression is subjective, and changes in aceto-whiteness and vascularity, which are not highly specific to HG–CIN, may include LG–CIN, inflammation, human papillomavirus infection and metaplasia, and performance between colposcopists is reported to be variable.^19^

In this study, sensitivity and specificity for colposcopy were calculated based on a contemporaneously recorded colposcopic impression for all biopsy sites. The only performance data collected from UK colposcopists is the PPV for colposcopic impression of HG–CIN, which should be >65%.^12^ The performance of colposcopy in this study was good: the lower 95% confidence interval for PPV exceeded 65%, and was compatible with published data.^7^,^8^,^10^,^11^,^20^ The prevalence of HG–CIN will affect the PPV of colposcopic impression for HG–CIN. In phase 2 of the study 43.7% of women were referred with high-grade cytology, and 52% of evaluable women were found to have HG–CIN on biopsy, and these rates are not dissimilar to disease rates in other studies.^8^,^9^,^20^,^21^ The referral rate with high-grade cytology was higher than the reported average for England (37%), reflecting the inclusion of ‘borderline–high grade not excluded’ cytology in the high-grade category.^13^

The primary objective of the study was to assess any improvement in the performance of colposcopy when using EIS as an adjunct. Phase 1 of the study demonstrated that the performance of EIS, using improved quality control and detection of tissue homogeneity, was significantly improved in comparison with our previously published results,^17^ with AUC increasing from 0.74 to 0.783 (*P* = 0.0024), demonstrating better separation of HG–CIN from non-HG–CIN. We chose to take EIS readings after the application of acetic acid in phase 2 of the study because it enhances the identification of the transformation zone, and EIS was unaffected by acetic acid.

We plotted an ROC curve for the performance of EIS as an adjunct to colposcopy to detect HG–CIN, and the area under the curve was significantly higher (0.887) than the figure of 0.82 quoted by Mitchell et al. and Cantor et al. when using colposcopy alone (*P* = 0.02).^7^,^11^ Caution needs to be exercised, however, when making this comparison as our ROC curve was calculated by varying the cut-off used on the CI + PI index, whereas Mitchell et al. generated an ROC curve by plotting the sensitivities and specificities reported in eight published reports of variable size and quality. It is not possible to create an ROC curve for colposcopic performance in a study such as ours, because colposcopic impression cannot be assessed as a continuous variable.

The specificity values that we report when using EIS as an adjunct, in comparison with colposcopic performance assessed either by the ability to detect high-grade disease (CI method) or by the ability to detect the presence of disease (DP method) and suggest points for biopsy, show significant increases: 7.3 and 26.6%, respectively. Similar significant increases in PPV are also shown: 8.4 and 13.5%, respectively. Although colposcopic impression is very relevant in assessing clinical performance at the time of examination, histology is the appropriate measure of the overall performance of colposcopy. The use of a high cut-off to increase specificity at the time of examination could be helpful when triaging women through colposcopy, and when treatment, based purely on colposcopy assessment, is being considered. Currently, treatment at first visit (‘see and treat’) is associated with an absence of HG–CIN on final histology in 23–59% of women.^13^,^16^ Women may therefore be put at risk of unnecessary morbidity, which incurs extra costs to the healthcare service; in addition, there is concern of a potential increased risk of premature labour in women who undergo excisional treatments.^22^–^24^ However, by increasing the cut-off applied to the CI + PI index to give a specificity of 95% the risk of over-treatment could be reduced. Many women are referred to colposcopy with low-grade cytology.^13^ Clinically it is important to identify within this group not only women who have HG–CIN but in addition women who have no significant disease. The use of EIS as an adjunct not only identified extra cases of HG–CIN in this group but also, through its higher specificity and negative predictive value, would allow colposcopists to return more women to routine recall, preventing repeat examinations.

The disease reference standards used in the study was defined as ‘histologically confirmed HG–CIN in any biopsy suggested either by the colposcopist or by EIS, where the EIS measurement exceeded the median for HG–CIN derived from phase 1’. Histology is the best available standard for the presence of HG–CIN. However, whereas histology is an adequate standard when a biopsy is taken, misclassification can arise where either no biopsy is taken or a biopsy is not taken where the disease is present in the cervix. Rutjes et al.2007 considered the question of inadequacies in reference or gold standards, and suggested ways of correcting a 2 × 2 table, where the probability of misclassifying false positives and true negatives is known. Based upon their report we would classify our reference standard as a ‘correct imperfect reference’. In our study there will be some women wrongly classified as non-HG–CIN because of under-sampling by biopsy. It is not possible to estimate the size of this group. However, we do know that the inclusion of EIS as an adjunct increased the number of women with HG–CIN biopsies. By definition these results appear in the TP group and were taken from the FP group, and hence will have increased PPV directly and also increased both sensitivity and specificity. Unfortunately, this does not enable us to estimate the likely changes to the accuracy parameters as a result of any remaining women wrongly classified as non-HG–CIN, because we do not know where these might appear in the 2 × 2 tables. However, this does not negate the improvement we have found in accuracy when EIS is used as an adjunct to colposcopy, because the same reference standard and patient group were used, both with and without the use of EIS.

Because of the ability to change the cut-off applied to the EIS measurement it is also possible to select a value that leaves specificity unchanged when using EIS as an adjunct. In this situation sensitivity, in comparison with clinical performance measured using the DP method (Table 3), was increased significantly from 88.5 to 96.6% (*P* = 0.006). The ability to increase sensitivity to detect disease is important if EIS is to be further evaluated as a screening test in different clinical scenarios. However, caution should be shown when extrapolating the results of the current study to the screening situation. The negative predictive values determined from the current colposcopy population with a high incidence of disease cannot be assumed to apply to a screening population with a low incidence of disease.

It might be argued that the use of EIS as an adjunct merely improves the true sensitivity because of the increased number of biopsies taken. Similar studies looking at adjuncts to colposcopy have reported biopsies per woman between 1.3 and 2.2, with the number of biopsies per woman related to the prevalence of suspected HG–CIN.^20^,^21^,^26^,^27^ In the ALTS trial 60% of women had two or more biopsies if they had a colposcopic impression of HG–CIN.^8^ Therefore, the biggest predictor of biopsies may be the index of suspicion rather than the technology. To assess if EIS increases the detection of HG–CIN by only detecting CIN2 we examined the ratio of CIN2 to CIN3 in the biopsies taken. The CIN2:CIN3 ratio in the UK published data is 1:1.75,^13^ in phase 2 the ratio was 1:1.77 and for HG–CIN detected by EIS alone it was 1:1.88, suggesting that disease detected by EIS was similar to the disease detected by colposcopists. To try to control for the impact of multiple biopsies the CI + PI method used to assess the performance of EIS was based upon only one biopsy per woman, with the biopsy being chosen on the basis of the highest PI at the time of examination. The improvement in performance is therefore based on a single biopsy for EIS compared with multiple biopsies taken by the colposcopist. In addition, the data in phase 2 were analysed on a per woman basis to reflect usual clinical practice, and women with HG–CIN on any biopsy will be managed differently from those who do not have HG–CIN. The improvements in performance using this type of analysis indicate that extra disease detected by EIS does not represent other foci of disease in women already known to have HG–CIN, but rather it detects HG–CIN in women who were thought not to have such disease based on colposcopic examination.

There are limitations to this study. Only three colposcopists used the device, but they represented a range of colposcopic experience, and once trained had little difficulty with its use. We did not review the cytology or histopathology because our objective was to evaluate the performance of the EIS device in routine clinical practice. It is widely acknowledged that there is both inter- and intra-observer variation between reporting pathologists; however, by using laboratories that take part in the relevant national screening quality assurance programmes, these variations will have been minimised to a standard that is acceptable for routine clinical use, hence the results of this study will be applicable to current colposcopy practice. It is possible that both colposcopy and EIS missed HG–CIN because we did not take random biopsies from normal quadrants of the cervix, or take biopsies from a specified part of the cervix if the colposcopic examination was normal; however, taking these biopsies is not part of routine clinical practice in the UK and Ireland. Data from several large UK and international studies have confirmed low rates of CIN2+ in women with negative colposcopic examination, suggesting that the value of single or four-quadrant random biopsies in routine practice would be minimal given the low PPV (<3%) for random biopsies in this situation.^14^–^16^,^26^,^28^ There is also a significant level of morbidity associated with the taking of cervical biopsies.^23^

We consider our understanding of the known changes in cellular and tissue structure associated with intraepithelial neoplasia to be a major strength of EIS, and, by comparing measured data with a defined model of tissues types, we can improve the accuracy of colposcopy in real time. The results of this study indicate that EIS alone performs similarly to colposcopy, but when used as an adjunct to colposcopy performance is significantly improved and fewer biopsies are required.

The EIS technology may have the potential for wider application. The facility to alter the sensitivity and specificity, along with the robustness of the device, suggests that EIS technology may have a role alongside tests such as visual inspection with acetic acid and primary human papillomavirus testing in low-resource settings.^29^ Given the histopathological similarities between intraepithelial neoplasia at other sites of squamous mucosal epithelia, EIS technology may have the potential to improve the diagnosis of pre-malignancies of the anus, oesophagus and vagina. We are currently evaluating the role of EIS in oropharyngeal disease.

## Conclusion

The use of EIS as a real-time adjunct to colposcopy improves clinical performance. The inclusion of EIS into colposcopic practice could lead to more appropriate patient management with lower intervention rates.

### Disclosure of interests

JAT and BHB hold patents related to the technology. They are shareholders in Zilico Ltd and receive consultancy fees. JH is a shareholder. WP is a medical advisor to Zilico Ltd and receives consultancy fees. HCK is Chair of the Advisory Committee for Cervical Screening, but the views expressed in this article do not represent the opinion of the Department of Health.

### Contribution to authorship

JAT co-wrote the study protocol, was chief investigator, performed colposcopic examinations, analysed the results and drafted the article. BHB co-wrote the study protocol, analysed the results and drafted the article. JH analysed the results and reviewed the article. SD performed colposcopic examinations and reviewed the article. MM performed colposcopic examinations and reviewed the article. WP reviewed the study protocol, was a co-investigator and reviewed the article. HCK reviewed the study protocol, was a co-investigator and drafted the article. The study design and protocol were written by JAT and BHB with input from WP and HCK Data collection and study monitoring was performed by Medvance Ltd. JAT, BHB, JH, SD and MM had access to the raw data and analysed the data.

### Details of ethics approval

This study was approved by Leeds (West) Medical Research Ethics Committee (ref. no. 08/H1307/80) on 28 August 2008 and Adelaide and Meath Hospital, Dublin, Research Ethics Committee on 11 January 2011.

### Funding

Zilico Ltd was the legal sponsor of the study, undertook regulatory and ethical approval, provided devices and tips, and contributed to data analysis. All authors had the final responsibility for the decision to submit for publication.
